# Increasing demand for ophthalmic pathology: time trends in a laboratory with nationwide coverage

**DOI:** 10.1186/s12886-023-02828-1

**Published:** 2023-03-06

**Authors:** Gustav Stålhammar, Emma Lardner, Marianne Georgsson, Stefan Seregard

**Affiliations:** 1 St. Erik Eye Hospital, Stockholm, Sweden; 2 Department of Clinical Neuroscience, Division of Eye and Vision, Karolinska Institutet, Stockholm, Sweden

**Keywords:** Ophthalmic pathology, Ocular pathology, Oncology, Time trend, Epidemiology, Population statistics

## Abstract

**Purpose:**

To report the time trends in basic patient characteristics and the number of specimens received at a national referral center for ophthalmic pathology.

**Methods:**

Data on patient sex, age at surgical resection and geographical location of the referring unit were obtained for all specimens received at the St. Erik Ophthalmic Pathology laboratory, Stockholm, Sweden, between January 1^st^, 1959, and December 31^st^, 2021.

**Results:**

A total of 33 057 specimens had been received, of which 14 560 (44%) came from men and 18 477 (56%) from women (for 20 patients, the sex was not specified). The average annual percent change (AAPC) in the number specimens received was + 10.5%, whereas the Swedish population increased with 0.5% per year. Patients became older throughout the period, with an average yearly increase of patient age at surgery of 0.3 years (AAPC 0.2%). Overall, women were three years older than men at surgery (59.4 versus 56.4 years, *P* < 0.0001) The number of specimens increased with patient age from the first to the 8^th^ decade, after which it decreased to zero in the 11^th^ decade. The largest portion of patients had undergone their surgery in one of the hospitals or clinics in the capital region, with four of the five largest sources corresponding to the most populous counties in the country.

**Conclusions:**

During six decades, the growth in number of specimens sent to our national referral center for ophthalmic pathology has greatly outpaced the growth of the population, indicating an increasing demand for subspecialized services. Throughout the period, patients have become older, and a higher number of specimens have been submitted from female patients.

## Introduction

The rise of pathology as an independent specialty more than 100 years ago was closely related to a fundamental driver of modern medicine: the urge to relate suffering and other symptoms to observable and measurable disease processes in their organs and cells [1]. In fact, the publication of *Die Cellularpathologie in ihrer Begründung auf physiologische und pathologische Gewebelehre* by Rudolph Virchow (who was not only a pathologist) in 1858 is regarded by many as the basis of modern medical science [2, 3]. In parallel, modern pathology was enabled by a technological evolution of specimen processing, staining procedures, the microtome and the light microscope with improved illumination, lenses and objectives in the end of the nineteenth century, by the likes of Lister, Abbe, Zeiss, Schott, Köhler, Cohnheim, von Recklinghausen, Strasburger and many others [4, 5]. During the twentieth century, the specialty evolved rapidly with the development of techniques such as phase contrast, differential interference contrast, immunofluorescence, immunohistochemistry, electron microscopy, polymerase chain reaction, gene sequencing and other techniques that have been elemental in our understanding of our own biology and have contributed greatly to man’s war on diseases [6, 7].

Ophthalmic pathology made its first steps towards a separate subspeciality in the United States in 1945 when American Ophthalmic Pathology Club (later named the Verhoeff Society and from 1985 the Verhoeff-Zimmerman Society) was organized at the Johns Hopkins University. Europe followed suite in with the inaugural meeting of The European Ophthalmic Pathology Club in London, 1962 [8].

In Sweden, ophthalmologist Albin Dalén pioneered ophthalmic pathology in 1904 with his studies of epithelioid cell accumulations in sympathetic ophthalmia, now known as Dalén-Fuchs noduli [9]. Von Hippel-Lindau syndrome is name after the Swedish pathologist Arvid Lindau who described the entity in 1926 [10]. In the 1950’s, pathologists at the Karolinska Hospital in Stockholm (known as the Karolinska University Hospital from 2004) started paying increasing interest to the eye and periocular areas. From 1959, all specimens from these anatomical locations were recorded in a dedicated registry, and in 1960 Erik Kock pioneered a separate ophthalmic pathology laboratory in the premises of the hospital [11]. In 1990, St. Erik Eye Hospital was founded and the laboratory moved to its building in central Stockholm and then followed suit with the relocation to Solna in 2020 -just across the road from the Karolinska University Hospital. Throughout the years, many ophthalmology centers in Sweden have not only sent their patients to use for treatment of ocular tumors, but also their excisions, biopsies, enucleations, and other types of specimens from the eyes and orbit to us for histopathological examination [12]. Virtually without exception, all the nation’s enucleated eyes with uveal melanoma and retinoblastoma, as well as transvitreal and transscleral biopsies of intraocular tumors, have been examined here. Similarly, a large share of all removed conjunctival melanomas, primary acquired melanosis (PAM), ocular surface squamous neoplasia (OSSN), and conjunctival squamous cell carcinomas are submitted to us. Some smaller specimens, depending on local routines and the need for assessment by an ocular pathologist, are instead examined by general pathologists, dermatopathologists, head&neck pathologists, or other pathologists with special interest in ocular pathology at the home hospitals. Eyelid specimens are commonly examined by dermatopathologists, although some of the largest institutions in the country refer these specimens to our laboratory. Full coverage of every ocular and periocular specimen in the country can therefore not be expected at our institution. However, as the ocular pathology laboratory has been the only one if its kind in the country since its foundation, the number of referred specimens should at least broadly reflect the demand for subspecialized ocular pathology in the country. Sixty years after its infancy, we therefore take the opportunity to review our ophthalmic pathology registry and the development of specimen volumes and patient sex and age characteristics over time.

## Method

Data on all specimens recorded in the dedicated Ophthalmic Pathology registry from its inception in 1959 through December 31^st^, 2021, were analyzed. If several specimens from the same patient had been received simultaneously, e.g., multiple biopsies taken at the same surgical session, they had been assigned the same case number and were counted as one specimen and one patient in the results. For each case in the registry, we had access to data on the year of the surgical procedure, clinic (i.e., hospital and city), patient sex, and patient age. No names, personal identification numbers, home addresses, contact information, photographs, or any other data that could be traced back to individual patients were collected. Further, no tissue specimens were analyzed in the project. According to the approved protocol (Swedish Ethical Review Authority, reference number 2022–00,930-02), the requirement for informed consent was waived considering that the study used no sensitive patient information; did not entail any treatment, other interventions, tests, examinations, or interviews; did not affect follow-up; did not expose patients to any risk of physical, psychological or other harm; and used no biological samples. The study adhered to the tenets of the Declaration of Helsinki.

### Statistical analysis

*P* values below 0.05 were considered statistically significant, all *P* values being two-sided. The number of specimens across patient age deviated significantly from normal distribution (Shapiro–Wilk test *P* < 0.05). For statistical comparisons of these two variables, we therefore used Kruskal–Wallis tests. No other continuous variable deviated significantly from normal distribution (Shapiro–Wilk test *P* > 0.05), why we used Student’s *t*-tests for these. In comparisons of parametrically distributed continuous variables between > 2 groups, we used one-way analysis of variance (ANOVA). In comparisons of categorical variables, we used two-by-two contingency tables and Pearson chi-square (χ^2^) tests (if all fields had a sample of > 5) or Fisher’s exact tests (if any field had a sample of < 5). For analysis of the number of yearly specimens as a function of time and patients’ age, we used curve estimations and tested if linear, logarithmic, quadratic, cubic or exponential models were best fitted to the data. In estimations of average annual percent change (AAPC) in the number of specimens received or patient age at surgery, we considered recommendations from the Surveillance Research Program at the National Cancer Institute [13]. We identified join points in the data and then calculated weighted average yearly percentual changes between these. A join point was defined as a high point or low point in the curve, which marked the beginning of an interval of at least 5 years with a change in the direction of the trend (i.e., from an increasing number of specimens to a decreasing, or vice versa). The yearly increase *y*_*1*_ in the number of specimens during a period of years *t*_1_ was calculated as:

$$n\mathrm{ specimens at the end of }{t}_{1} = n\mathrm{ specimens in the beginning of }{t}_{1} \times {{y}_{1}}^{{t}_{1}}$$

For example: If we received 10 specimens in 1980 and 100 specimens in 1985, the yearly increase *y* was 1.58 (or 58%), calculated as 100 = 10 × *y*^5^. Naturally, this rate is not necessarily representative of the whole period 1959—2021. The AAPC during the entire period consisting of several such periods separated by join points (for example *t*_1_, *t*_2_ and *t*_3_) is calculated as:

$$AAPC= \frac{\left({y}_{1}\times {t}_{1}\right)+\left({y}_{2}\times {t}_{2}\right)+{\left({y}_{3}\times {t}_{3}\right)}}{({t}_{1}+{t}_{2}+{t}_{3})}$$

Weighted averages of positive and negative *y* were calculated separately and then added together. This method takes more data points than just the first and last value in the series into account, including temporary deviations from the general trend [14]. The trend for number of specimens per year was compared with the size of the Swedish population using data from Statistics Sweden [15]. The mean age at surgery over time was compared to life expectancy for Swedish men and women at birth from the United Nations Population Division [16]. All statistical analyses were performed using IBM SPSS statistics version 27 (Armonk, NY, USA). Maps were made with mapchart.net under a Creative Commons Attribution-ShareAlike 4.0 International License.

## Results

### Descriptive statistics

A total of 33 057 specimens were sent to the ocular pathology service between January 1^st^, 1959, and December 31^st^, 2021. These specimens had been surgically removed from 14 560 men (44%) and 18 477 women (56%). For 20 patients (< 1 ‰), the sex was unknown. The mean patient age at surgery was 58 years (SD 23, Table 1). One hundred and forty-seven patients were less than 1 year old at surgery, and 41 patients were older than 100 years. The oldest patients were a trio of ladies aged 104 years.

**Table 1:** Patient characteristics

***n***	33 057
**Sex, *n* (%)**	
Men	14 560 (44)
Women	18 477 (56)
N/a	20 (< 1 ‰)
**Age at surgery, mean years (SD)**	58 (23)
**Age at surgery, n **	
0—9 years	1515
10—19 years	1406
20—29 years	1575
30—39 years	2275
40—49 years	2949
50—59 years	4206
60—69 years	5589
70—79 years	6924
80—89 years	5231
90—99 years	1301
100— years	62
N/a	24

N/a, Not available. *SD* Standard deviation

### Number of specimens per year

During 1959 through 1967, less than 100 specimens were received per year. The number of yearly specimens then increased to > 1 000 from 2011 and onwards, except for 2012, 2013 and 2015 in which 944 to 946 specimens were received.

**Fig. 1:**
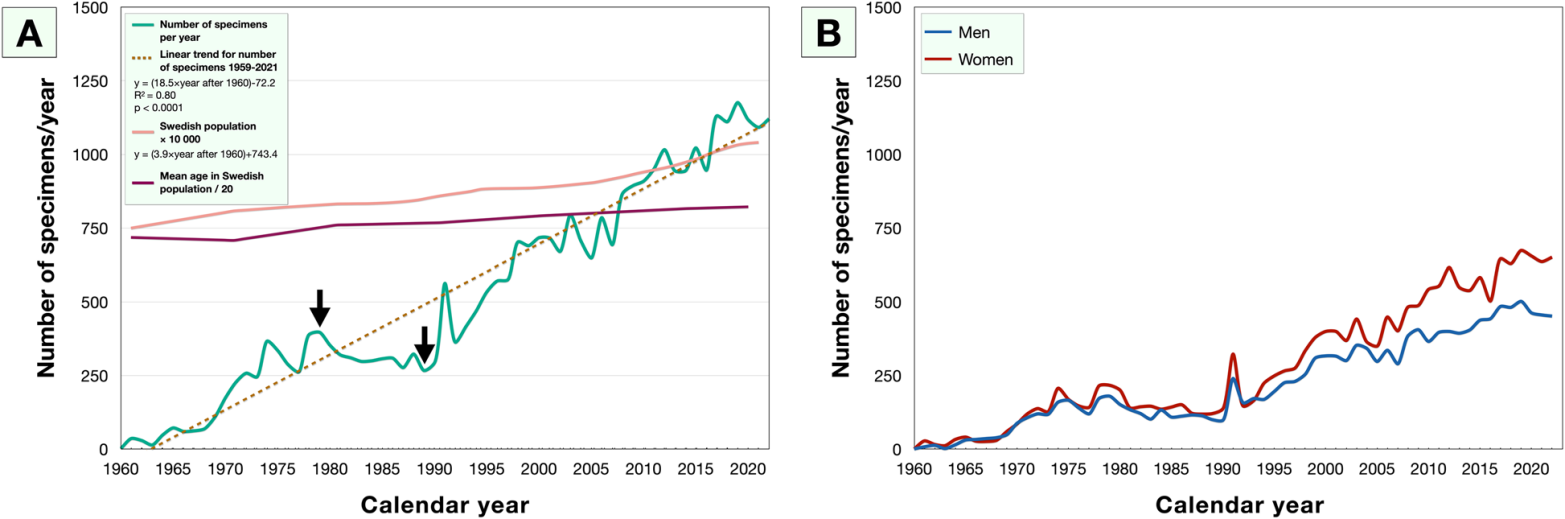
Number of specimens received during the period 1959 to 2021. A) The number of specimens received increased from *n* = 2 in 1959 to *n* = 1121 in 2021. The average annual percent change (AAPC) for the entire period was + 10.5%. This can be compared with the size of the Swedish population, which increased with an AAPC of 0.5% during the same period (pink line) and with the mean age in the Swedish population, which increased with an AAPC of 0.2% (purple line). In linear regression analysis, the number of specimens increased with a slope coefficient of 18.5 per year (brown dashed line). Arrows indicate join points in the data. B) In every single year except 1965—1967, 1969, 1991 and 1992 we received more specimens from female (red) than male patients (blue)

Two join points were identified in the curve: After receiving an increasing number of yearly specimens from 1959 to 1978, numbers decreased until 1988 after which they again started a long trend of increasing numbers until present date. The AAPC for the entire period was + 10.5%. This can be compared with the size of the Swedish population, which increased with an AAPC of 0.5% per year during the same period (Fig. 1A). In linear regression analysis, the number of specimens increased with a slope coefficient (β) of 18.5 per year after 1959. The number of specimens per year fit to a linear function (R^2^ = 0.80, *P* < 0.0001), where *x* was the consecutive year after 1959: Number of specimens = (18.5*x*) – 72.2. Thus, if the long-term trend continues, 1 241 cases will be received in year 2030 ((18.5 × 71) – 72.2) and 1 611 cases in year 2050.

In every single year except 1965—1967, 1969, 1991 and 1992 we received more specimens from female than male patients (Fig. 1B). On a year-per-year basis, the number of specimens from females were significantly higher than from males (Fisher’s exact *P* < 0.0001).

### Age at surgery

**Fig. 2:**
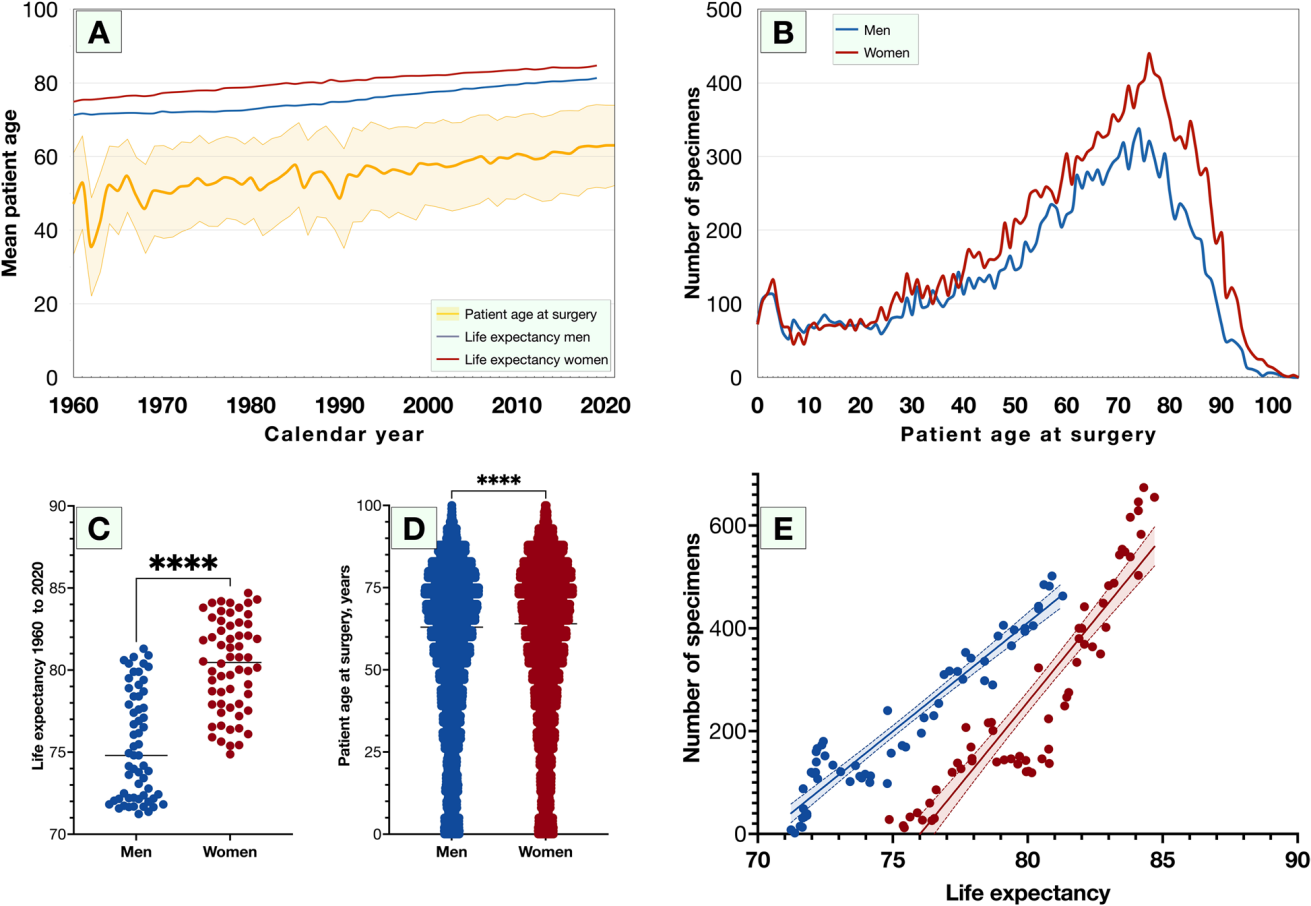
Patient age and life expectancy during the period 1959 to 2021. A) Mean age for all patients (yellow line, surrounding yellow field indicates one standard deviation). The annual increase of patient age at surgery was 0.3 years, corresponding to an AAPC of 0.5%. This can be compared with the life expectancy at birth for men (blue line) and women (red line), which increased with in average 0.2 years (AAPC 0.2%) yearly for both sexes during the same period. B) Number of men (blue) and women (red) of age 0—104 years. C) On average, the life expectancy was five years longer for women throughout the period. D) Women were three years older than men at the time of surgery. E) In linear regression, the number of received specimens received was a function of life expectancy for men (β = 42.3, R^2^ = 0.91, *P* < 0.0001) and women (β = 64.4, R^2^ = 0.85, *P* < 0.0001). ****, significant on the 0.0001 level

Patients became older throughout the period 1959—2021 (β = 0.3, R^2^ = 0.46, *P* < 0.0001, Fig. 2A). The annual increase of patient age at surgery was 0.3 years, corresponding to an AAPC of 0.5%. This can be compared with the life expectancy for men and women, which increased with in average 0.2 years (AAPC 0.2%) yearly for both sexes during the same period, or with the mean age of the Swedish population which increased with in average 0.09 years (AAPC 0.2%) yearly. On average, the life expectancy at birth was five years longer for women throughout the period (80.3 years versus 75.4 years, Student’s *t*-test *P* < 0.0001), and women were three years older than men at the time of surgery (59.4 years versus 56.4 years, Student’s *t*-test *P* < 0.0001, Figs. 2B to 2D). In linear regression, the number of received specimens received was a function of life expectancy for men (β = 42.3, R^2^ = 0.91, *P* < 0.0001) and women (β = 64.4, R^2^ = 0.85, *P* < 0.0001, Fig. 2E). Further, the number of specimens increased for every decade of patients’ age from the first to the 8^th^ decade, after which it decreased to zero in the 11^th^ decade. The curve was best described by a cubic function (R^2^ = 0.88, *P* < 0.0001), where *x* was the age of the patient (in years): Number of specimens =  − 0.006*x*^3^ + 0.8*x*^2^ − 21.8*x* + 277.3. Due to this arched curve, the number of specimens was similarly distributed over year or decade of patients’ age (Kruskal–Wallis *P* = 0.48 and 0.43, respectively).

### Enucleations for uveal melanoma and retinoblastoma

**Fig. 3:**
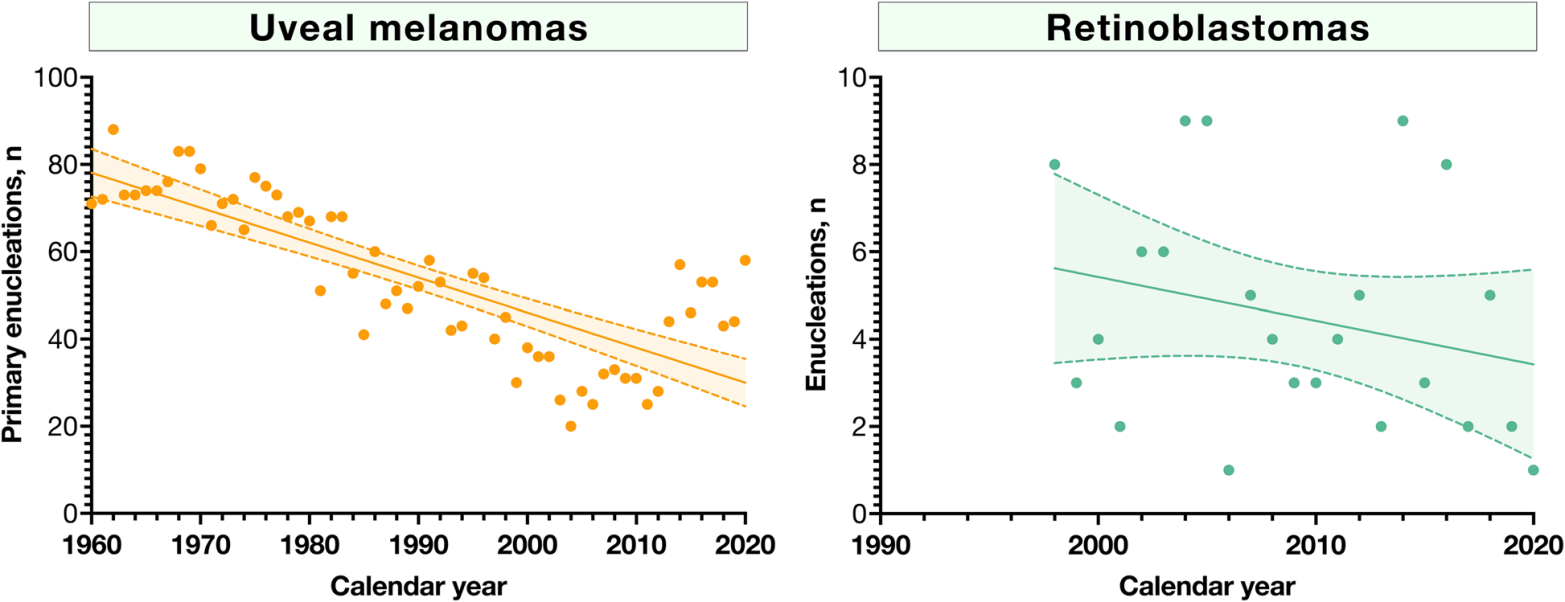
Enucleations for uveal melanoma and retinoblastoma. A) In linear regression, enucleation became a less frequent primary treatment for uveal melanoma during the period (β = -0.8, R^2^ = 0.64, *P* < 0.0001). B) There was no significant trend for the number of children that underwent enucleation for retinoblastoma between 1998 and 2020 (β = -0.1, R^2^ = 0.07, *P* = 0.23)

Enucleation was the only treatment modality offered for primary uveal melanomas in Sweden until 1979, in which brachytherapy with Ruthenium-106 was introduced. The share of patients treated with primary brachytherapy increased thereafter. In 1999, brachytherapy with iodine-125 was introduced and is still generally reserved for tumors with an apical height of about 6 to 10 mm [17]. Enucleation is still a widely used treatment alternative and we currently receive about 50 to 60 specimens a year. Assuming a population of currently 10 million and a crude incidence rate of 9.5, this represents 53 to 63% of newly diagnosed uveal melanomas in the country. In linear regression, enucleation became a less frequent primary treatment for uveal melanoma during the period (β = -0.8, R^2^ = 0.64, *P* < 0.0001). However, there was no significant trend for the number of children that underwent enucleation for retinoblastoma during the period with available data (1998 to 2020, β = -0.1, R^2^ = 0.07, *P* = 0.23, Fig. 3).

### Geographical location

Data on location of surgery were available for 21 636 patients (65%). The largest portion of patients had undergone their surgery in one of the hospitals or clinics in Stockholm (14 985, 45%). The second, third, fourth and fifth largest sources were Uppsala (2 184, 7%), Göteborg (1 988, 6%), Visby (632, 1.9%) and the combination of Malmö and Lund (321, 1.0%), respectively. All other locations had sent < 1% of the specimens (Table 2).

**Table 2:** Location of surgery by town/city

Site	*n*	%
Borås	47	0.1
Eksjö	6	0.0
Eskilstuna	38	0.1
Falun	48	0.1
Gällivare	1	0.0
Gävle	17	0.1
Göteborg	1 988	6.0
Halmstad	111	0.3
Härnösand	1	0.0
Helsingborg	55	0.2
Hudiksvall	16	0.0
Jönköping	108	0.3
Kalmar	37	0.1
Karlskrona	25	0.1
Karlstad	29	0.1
Katrineholm	1	0.0
Kristianstad	10	0.0
Landskrona	1	0.0
Linköping	146	0.4
Lund	251	0.8
Malmö	70	0.2
Norrköping	18	0.1
Nyköping	4	0.0
Örebro	92	0.3
Örnsköldsvik	8	0.0
Östersund	26	0.1
Piteå	2	0.0
Skellefteå	1	0.0
Skövde	95	0.3
Sollefteå	4	0.0
Stockholm, all hospitals	14 985	45.3
Sunderby	37	0.1
Sundsvall	35	0.1
Trollhättan	23	0.1
Uddevalla	82	0.2
Umeå	145	0.4
Uppsala	2 184	6.6
Västerås	99	0.3
Västervik	20	0.1
Växjö	103	0.3
Visby	632	1.9
International	35	0.1
N/a	11 421	34.5
**Total**	**33 057**	100.0

**Fig. 4:**
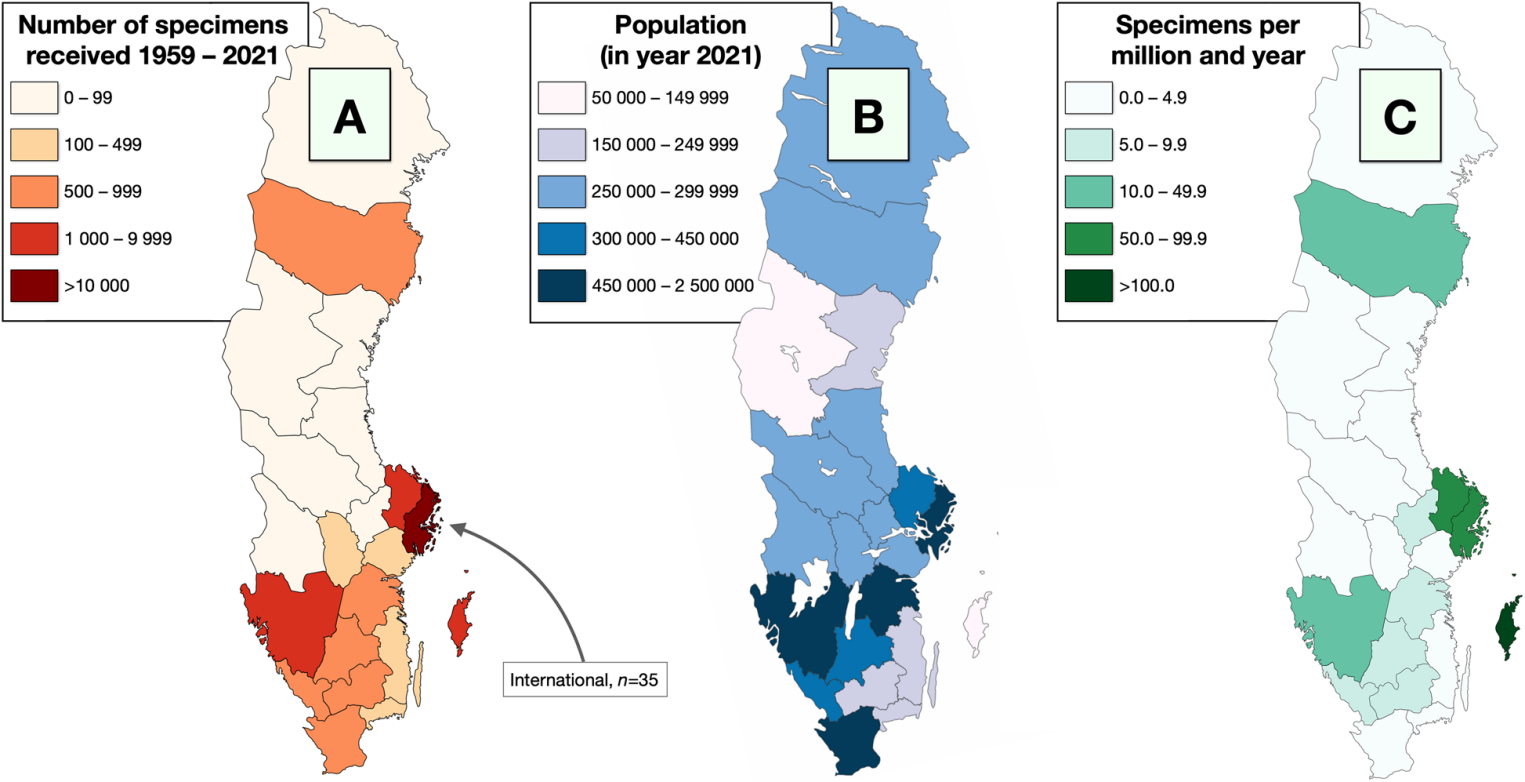
Geographical origin of the specimens sent to the laboratory during the period 1959 to 2021. A and B) The majority of all samples came from the most populous counties in the country. C) Illustrated as the number of submitted specimens per million inhabitants and year, the island of Gotland, and the capital regions were overrepresented. Thirty-five specimens were submitted from abroad. The maps were made with mapchart.net under a Creative Commons Attribution-ShareAlike 4.0 International License

During the period, we have received specimens from all administrative regions in Sweden with the distribution roughly following the population density in the country (Table 3). In terms of the number of specimens received per million inhabitants and year, the island of Gotland, and the Stockholm and Uppsala regions were overrepresented (Fig. 4). In linear regression, the number of specimens received correlated to county population (R^2^ = 0.55, *P* < 0.0001).

**Table 3:** Location of surgery by county

Site	*n*	%
Blekinge	26	0.1
Jämtland	26	0.1
Värmland	29	0.1
Gävleborg	33	0.1
International	35	0.1
Norrbotten	40	0.1
Södermanland	43	0.1
Västernorrland	48	0.1
Dalarna	48	0.1
Kalmar	57	0.2
Örebro	92	0.3
Västmanland	99	0.3
Kronobergs län	103	0.3
Halland	111	0.3
Jönköping	114	0.3
Västerbotten	146	0.4
Östergötland	164	0.5
Skåne	386	1.2
Gotland	632	1.9
Uppsala	2184	6.6
Västra Götaland	2235	6.8
Stockholm	14,985	45.3
International	35	0.1
N/a	11 421	34.5
**Total**	**33 057**	**100**

## Discussion

In this study, we have found that the number of specimens sent to our ophthalmic pathology laboratory has been steadily increasing since its infancy in 1959. During the period 1978 – 1988, the volumes temporarily decreased. The overall trend is however very clear: We now receive > 1 000 samples per year which is more than threefold the rate in any year prior to 1990, and this increase cannot be dismissed as solely the result of the slowly increasing size of the Swedish population. Our patients have become older along with the rest of the population, and an older population is more likely to develop many of the tumors and other conditions we diagnose [18, 19]. Increasing age is therefore a plausible factor driving at least a portion of the increased demand on our services. In turn, the longer life expectancy of women and their significantly older mean age is a likely reason for their overrepresentation in the current data. However, as indicated by the 50-fold higher AAPC of the number of specimens received than the AAPC of the mean age of the Swedish population, the contribution of the latter to the former is likely small. The sex disparity may also be caused by a greater tendency of women to seek the attention of health care services including diagnostic procedures, as observed previously [20]. This would however not explain the great increase in the number of specimens over time. We rather believe that the prime driver of the rising number of specimens is increasing demand for the specific competence and experience of dedicated ophthalmic pathologists among ophthalmologists and fellow pathologists. This would be in line with the general development in medicine with increasing subspecialization and complexity to the point were few generalists remain [21, 22].

The number of received specimens temporarily declined from 389 in 1977 to 221 in 1988. This period coincided with the introduction of plaque brachytherapy for uveal melanoma in 1979, with a decreasing proportion of patients being treated with primary enucleation during the 1980s. Other possible reasons for the period with smaller volumes could be related to changes in healthcare practices and tendencies of ophthalmologists to submit their specimens. In contrast to recommendations for pathological examination of virtually all tissue that is removed from patients in the current day, individual surgeons had a higher influence on the decision whether a piece of tissue should be submitted or not in the period before 1990.

Further, between different counties, the number of received specimens varied between 1 and 167 per million inhabitants and year. This broad range is unlikely to represent true differences in the distribution of ocular diseases between the counties. For example, the capital region was highly represented in the number of specimens, but is characterized by a relatively young and wealthy population, with relatively good access to ophthalmologists. Our interpretation is therefore that this variance is rather primarily associated with differences in healthcare availability. Despite having a single-payer system with efforts to offer equal healthcare to all, the access to both primary health care and ophthalmologists is unequal throughout the country, due to difficulties in manning all positions, differences socioeconomic factors, geographical distances, and local policies [23–26].

The demand for ophthalmic pathology could very well have been measured in other units than the number of specimens sent to our laboratory, such as the time or resources spent on each individual specimen. Previous studies indicate that pathologists’ workload increase over time even without increasing specimen volumes, due to a constantly increasing number of available analyses, treatment options and expectations on the amount of prognostic information that can be extracted from the examination of a lesion [27, 28]. For example, it is no longer sufficient for the pathologist to just state a diagnosis, a size of a lesion and the excision margins based on the examination of a tissue sample. It is also expected that more detailed information can be provided, e.g., if a specimen contains a more benign or more aggressive variant of the disease (prognostic information) and, if available, what treatments may be effective (predictive information). In other words, pathologists cannot simply state that a specimen contains a melanoma, inflammation, or other entity, but must declare what type of melanoma or inflammation it is, what growth pattern it has or what mutations or protein expression patterns have been found (e.g., *BRAF* mutation in eyelid melanoma, *BAP1* mutation or BAP-1 protein expression in uveal melanoma and IgG4-positivity in inflammation etc.). In a report from a large German institute of pathology from 2016, Warth et al. found that the number of slides per case had increased with 60% during the previous decade, that the number of immunohistochemistry procedures had doubled and that the number of molecular analyses had more than tripled [29]. This development is set to continue in its current direction.

The strengths of this study include the large and complete data set of all received specimens during more than 60 years. Throughout the period, the St. Erik Ophthalmic Pathology laboratory and its predecessor has been the only laboratory of its kind in Sweden. Enucleated eyes with uveal melanoma and retinoblastoma, and biopsies of intraocular tumors, have been examined by us exclusively. Further, the availability of additional data such as patient age, sex, and geographical origin allows for important analyses of some characteristics, trends, and possible confounders. Swedish healthcare has a similar organization as the other Nordic countries, with similar life expectancy and burden of disease, and in the light of a similar development over time in other subspecialities in the western world, we think our results give a fair representation of an increasing demand of our subspeciality [21, 22, 27–30].

The present study also has several limitations. Firstly, it should be stressed that the herein presented data should be regarded as an overview of the number of specimens received and of the age and sex of the patients from which these specimens were removed. The data do not make room for more detailed analyses. We interpret the increasing number of specimens as an increased demand for ophthalmic pathology, be the causality cannot be determined with the available information and the increase may hypothetically be due to several reasons not discussed here. Secondly, the data presented must not be interpreted as a complete account of every specimen removed from the eyes and periocular region in the country during the period. Even though our laboratory is the only dedicated to ophthalmic pathology, an unknown number of specimens has been examined at other hospitals at the discretion of the referring clinician and local routines. Thirdly, we had no access to data on tissue diagnoses for the received specimens, which would have added significant value to this article. The exception was data on the number of enucleations for uveal melanoma, which were relatively uncomplicated to collect as this diagnosis is associated closely related to this type of specimen. Lastly, even we counted multiple simultaneous specimens from the same patient as one specimen from one patient, it is still possible that the same patient occurred more than once in the data, e.g., if a patient removed an eyelid tumor and then a recurrence five years later. The reported number of specimens may therefore not represent an identical number of individual patients.

In conclusion, our ophthalmic pathology laboratory has received an increasing number of yearly specimens from gradually older patients since its infancy in 1959. Although most specimens have been received from the vicinity of the laboratory, all administrative regions in Sweden have sent specimens to us, with the distribution roughly following the population density in the country. Throughout the period, we have received more specimens from women, which may be a result of their longer life expectancy. Future studies may aim to investigate the reasons for the increasing demand on our services.

## Data Availability

The data that support the findings of this study are available from the corresponding author upon reasonable request.
